# Patient demographics, clinicopathologic features, and outcomes in wild-type gastrointestinal stromal tumor: a national cohort analysis

**DOI:** 10.1038/s41598-022-09745-1

**Published:** 2022-04-06

**Authors:** Tahsin M. Khan, Emily A. Verbus, Alexander J. Rossi, Jonathan M. Hernandez, Jeremy L. Davis, Brian A. Coakley, Andrew M. Blakely

**Affiliations:** 1 Surgical Oncology Program, National Cancer Institute, Bethesda, MD USA; 2 Department of Surgery, The Mount Sinai Hospital, New York, NY USA; 3 Surgical Oncology Program, National Cancer Institute, 9000 Rockville Pike Building 10, Room 4-3760, Bethesda, MD 20892 USA

**Keywords:** Surgical oncology, Oncology, Cancer, Cancer genetics, Gastrointestinal cancer, Oncogenes

## Abstract

Wild-type *KIT* and *PDGFRA* gastrointestinal stromal tumors (GIST) are rare tumors with limited treatment options. We sought to determine the clinicopathologic features of wild-type GIST and identify factors that influence overall survival (OS) using a large national database. Retrospective evaluation of patients with wild-type GIST in the National Cancer Database (NCDB) was performed. Demographic, clinicopathologic, and treatment data were analyzed. Features associated with OS were investigated using Kaplan–Meier analysis and Cox proportional hazards model. 244 patients with median diagnosis age of 59 years (95% CI 57–63) were identified. The stomach was the most common primary site (57%) followed by the small intestine (35%). Surgical resection was performed on 85% of patients and 53% of patients received systemic therapy. Factors associated with decreased OS on multivariable analysis included small intestine primary (HR 2.72, 95% CI 1.13–6.69, *P* = 0.026) and > 5 mitoses per 50 HPF (HR 4.77, 95% CI 1.86–13.2, *P* = 0.001). Wild-type GISTs may be identified in older patients, with most arising in the stomach and small bowel. Surgery remains the principal treatment modality. Small intestine primary site and high mitotic count were associated with abbreviated OS.

## Introduction

Most gastrointestinal stromal tumors (GIST) harbor mutations in either the KIT proto-oncogene (*KIT* [OMIM 164920]; 80%) or platelet-derived growth factor receptor a (*PDGFRA* [OMIM 173490]*;* 15%) receptor tyrosine kinases^1–3^. However, up to 10% of GIST are found to have non-mutated *KIT* and *PDGFRA* and are therefore called “wild-type” GIST (WT GIST)^3,4^. The drivers of tumorigenesis in these lesions are diverse and include alterations to *SDHx* genes (subunits A [OMIM 600857], B [OMIM 185470], C [OMIM 602413], or D [OMIM 602690]), RAS pathway genes such as neurofibromin 1 (*NF1* [OMIM 613113]), B-Raf proto-oncogene serine-threonine kinase (*BRAF* [OMIM 164787]), or K-Ras proto-oncogene GTPase (*KRAS* [OMIM 1900070]), or alterations in other receptor tyrosine kinases such as neurotrophic tyrosine kinase receptor Type 1 and 3 (*NTRK1* [OMIM 191315], *NTRK3* [191316])^3,5–8^. Unlike *KIT/PDGFRA* mutation-driven GIST, WT GIST are often not responsive to targeted therapy with the first-generation tyrosine kinase inhibitor (TKI) imatinib, and only limited clinical benefit has been observed with the newer-generation TKIs sunitinib and regorafenib^9–11^. Other experimental therapies have been found to be either ineffective or overly toxic. Therefore, surgery remains the mainstay of treatment for patients with WT GIST^4^.

To date, the largest accumulated experience in managing patients with WT GIST has been through the United States National Institutes of Health’s WT GIST Clinic^4^. The clinic was started in 2008 to provide expert, multidisciplinary management recommendations for this rare tumor. Although medical care was provided by patients’ local physician teams, retrospective analysis of demographic data collected by this clinic established that patients with WT GIST were predominantly female with a median age of presentation of 21 years^4^. This finding furthered the dogma that WT GIST primarily affects younger patients. The disease course was characterized as indolent yet relentless, and surgical resections after removal of the primary were discouraged given the observation of relatively rapid recurrence rates^4^. Aside from this single report from the NIH Pediatric and Wild-Type GIST Clinic, the only other large-scale characterization of adolescent and young adult (AYA) patients with GIST was a recent National Cancer Institute Surveillance, Epidemiology, and End Results (SEER) database analysis^12^. This analysis found that surgical resection, even in the setting of metastatic disease, was associated with improved survival. However, this analysis did not specifically focus on patients with wild-type GIST, and molecular characteristics of the tumors could not be precisely ascertained given lack of capture by the SEER database^12^.

A national-level analysis of WT GIST has not been performed, and existing studies are limited by referral bias. In this context, the National Cancer Database (NCDB) offers a unique opportunity to study a large, heterogenous cohort of patients with WT GIST given its scale, documentation of granular pathological data including *KIT* and *PDGFRA* mutation status, defined treatment patterns including administration of surgery and systemic therapy, and long-term follow-up. In evaluating the NCDB, we endeavored to expand upon the aforementioned NIH WT GIST Clinic and SEER database analyses by focusing on a national cohort of patients with GIST with documented wild-type *KIT* and *PDGFRA*. We aimed to determine the clinicopathologic features of this tumor type and identify the factors that influenced overall survival in order to provide a more complete understanding upon which management recommendations could be based for this rare tumor.

## Results

Overall, 244 patients with GIST harboring wild-type *KIT* and *PDGFRA* were identified (Table 1). Patients were predominantly male and Caucasian, with private insurance or Medicare. The median age at diagnosis was 59 years. The primary site was most frequently stomach (n = 139, 57.0%), followed by small intestine (n = 86, 35.2%), colon (n = 8, 3.3%), esophagus (n = 5, 2.0%), rectal (n = 5, 2.0%), and appendix (n = 1, 0.4%). Tumors were frequently greater than 5 cm in diameter (43%), whereas few were 2 cm in size or less (5%). Mitotic rate was well-captured in the database, with most specimens demonstrating up to 5 mitoses per 50 HPF (66%). Most patients underwent surgical resection (85%), and of these most tumors were removed with negative margins (83.7%). Of the 23 patients with positive tumor margins, 12 (52%) were microscopically positive, 2 (9%) were macroscopically positive, and the remainder were not otherwise specified. Over half of the patients were given systemic therapy (53%). 10 patients (4.1%) we documented to have > 2 tumors during their lifetime, suggesting the presence of hereditary syndromic disease. There were no differences in demographic, clinicopathologic, or treatment variables across primary sites. Similarly, a focused analysis of only patients with stomach or small intestine primaries yielded no significant differences between the groups, aside from a significantly higher proportion of patients who did not undergo surgical resection among the gastric patients (17% versus 10%, p = 0.040) (Table S1).

**Table 1:** Patient demographics, clinicopathologic characteristics, and treatment characteristics, overall and by primary site.

		Total n = 244 N (%)	Stomach n = 139 N (%)	Small Intestine n = 86 N (%)	Other n = 19 N (%)	*P* value
**Patient characteristics**						
Age at diagnosis	< 18	8 (3)	8 (6)	–	–	0.19
	18–40	36 (15)	23 (17)	11 (13)	2 (11)	
	40–65	112 (46)	59 (42)	45 (52)	8 (42)	
	> 65	88 (36)	49 (35)	30 (35)	9 (47)	
Sex	Male	136 (56)	77 (55)	49 (57)	10 (53)	0.94
	Female	108 (44)	62 (45)	37 (43)	9 (47)	
Race/ethnicity	White	174 (71)	92 (66)	67 (78)	15 (79)	0.55
	Black	27 (11)	18 (13)	7 (8)	2 (11)	
	Hispanic	29 (12)	19 (14)	8 (9)	2 (11)	
	Other	14 (6)	10 (7)	4 (5)	-	
Insurance	Private	124 (51)	74 (53)	41 (48)	9 (47)	0.47
	Medicare	86 (35)	44 (32)	34 (40)	8 (42)	
	Medicaid	14 (6)	6 (4)	7 (8)	1 (5)	
	Other	20 (8)	15 (11)	4 (5)	1 (5)	
Charlson comorbidities	None	184 (75)	109 (78)	61 (71)	14 (74)	0.59
	1	45 (18)	24 (17)	17 (20)	4 (21)	
	≥ 2	15 (6)	6 (4)	8 (9)	1 (5)	
**Clinicopathologic characteristics**						
Size	≤ 2 cm	13 (5)	8 (6)	3 (3)	2 (11)	0.75
	2.1 to 5 cm	59 (24)	31 (22)	24 (28)	4 (21)	
	> 5 cm	105 (43)	64 (46)	33 (38)	8 (42)	
	Tx	67 (27)	36 (26)	26 (30)	5 (26)	
Grade	Low	54 (22)	29 (21)	22 (26)	3 (16)	0.91
	Moderate	43 (18)	24 (17)	16 (19)	3 (16)	
	High	18 (7)	10 (7)	7 (8)	1 (5)	
	Unknown	129 (53)	76 (55)	41 (48)	12 (63)	
Mitoses	≤ 5 per 50 HPF	162 (66)	87 (63)	63 (73)	12 (63)	0.27
	> 5 per 50 HPF	52 (21)	32 (23)	17 (20)	3 (16)	
	Not specified	30 (12)	20 (14)	6 (7)	4 (21)	
Lymph-vascular invasion	Absent	82 (34)	51 (37)	27 (31)	4 (21)	0.27
	Present	7 (3)	6 (4)	1 (1)	-	
	Unknown	155 (64)	82 (59)	58 (67)	15 (79)	
Multifocality	Absent	184 (75)	104 (75)	63 (73)	17 (89)	0.27
	Present	38 (16)	21 (15)	17 (20)	-	
	Unspecified	22 (9)	14 (10)	6 (7)	2 (11)	
Node status	Negative	85 (35)	42 (30)	35 (41)	8 (42)	0.21
	Positive	15 (6)	12 (9)	3 (3)	-	
	Unknown	144 (59)	85 (61)	48 (56)	11 (58)	
Metastasis at diagnosis	Absent	208 (86)	114 (83)	78 (92)	16 (84)	0.16
	Present	34 (14)	24 (17)	7 (8)	3 (16)	
**Treatment characteristics**						
Surgical margins	Negative	174 (71)	104 (75)	59 (69)	11 (58)	0.13
	Positive	23 (9)	7 (5)	13 (15)	3 (16)	
	Unknown	11 (5)	5 (4)	5 (6)	1 (5)	
	Surgery not performed	36 (15)	23 (17)	9 (10)	4 (21)	
Systemic therapy	No therapy given	111 (46)	66 (47)	37 (43)	8 (42)	0.77
	Therapy received	130 (53)	72 (52)	47 (55)	11 (58)	
	Unknown	3 (1)	1 (1)	2 (2)	-	

*HPF* high-power field.

A subset analysis of the 139 patients with gastric primary GIST was performed to better delineate the primary tumor location within the stomach. Of the 89 patients who had a discrete tumor location indicated, 23 (26%) originated from the proximal stomach, 48 (54%) in the body, and 18 (20%) in the distal stomach; there was no association between location or any patient demographic, clinicopathologic, or treatment variable (data not shown). Overall, 116 gastric resections were performed comprising 103 (89%) subtotal gastrectomies, 10 (9%) wedge resections, 2 total gastrectomies, as well as one resection not otherwise specified.

A second subset analysis of patients with small bowel GIST was performed by stratifying duodenal versus more distal location. Of the 60 intestinal primary tumors with documented location, 27 (45%) were located in the duodenum. Duodenal primaries were more likely to be diagnosed at a tumor size ≤ 5 cm than jejunoileal primaries (68% vs. 31%, p = 0.043). No other associations between tumor location and patient demographic, clinicopathologic, or treatment variables were identified (data not shown).

**Figure 1:**
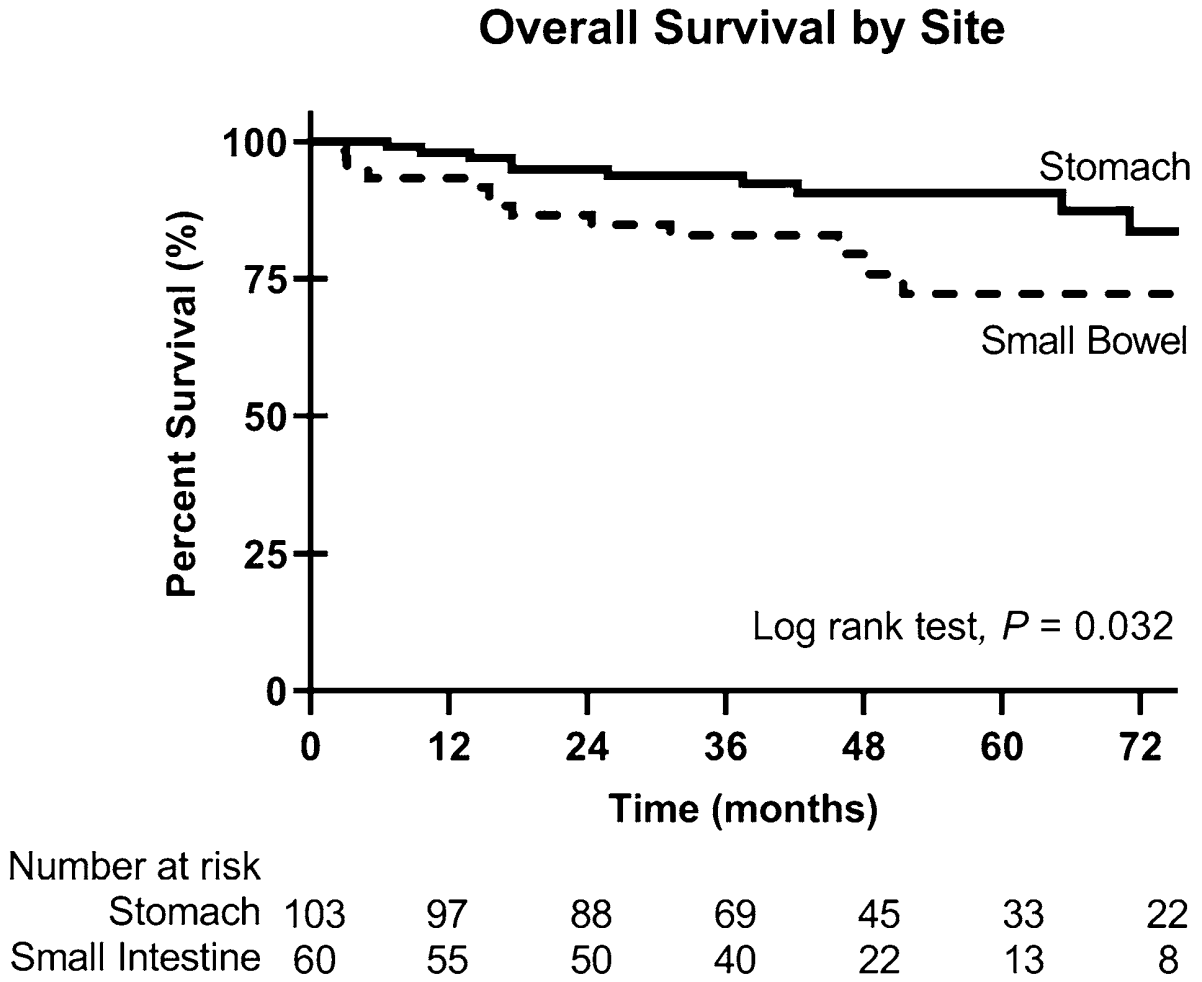
Kaplan–Meier curves for overall survival in gastric and small bowel WT GIST. Small bowel primary site is significantly associated with abbreviated overall survival compared to gastric primary.

Overall survival (OS) analysis was restricted to stomach and small intestine primary tumors (Table 2). On univariate analysis, small intestine primary site, larger tumor size, increased mitotic rate, and systemic therapy administration were associated with decreased OS. Of note, patient age, sex, tumor multifocality, lymph node involvement, and metastatic disease on diagnosis were not associated with OS. On multivariable analysis, small intestine primary site and > 5 mitoses per 50 HPF were independently associated with decreased OS. Kaplan–Meier curves of OS stratified by primary site were constructed, with neither curve reaching median survival but demonstrating significantly worse OS for patients with small intestine primary tumors (log-rank p = 0.032) (Fig. 1).

**Table 2:** Univariable and multivariable Cox proportional hazards analyses of patient demographic, clinicopathologic, and treatment factors, with overall survival AS outcome.

		N (%)	Univariable analyses				Multivariable analysis			
			HR		95% CI	*P* value	HR		95% CI	*P* value
**Patient characteristics**										
Age at diagnosis	< 40	36 (22)	Ref		–	–	–		–	–
	41–65	78 (48)	1.27		0.43–4.57	0.68				
	> 65	49 (30)	1.41		0.44–5.27	0.57				
Sex	Male	84 (52)	Ref		–	–	–		–	–
	Female	79 (48)	0.73		0.31–1.67	0.46				
Race/ethnicity	White	113 (69)	Ref		–	–	–		–	–
	Black	17 (10)	0.94		0.15–3.41	0.93				
	Hispanic	24 (15)	2.19		0.77–5.56	0.13				
	Other	9 (6)	2.79		0.43–10.2	0.24				
Insurance	Private	92 (56)	Ref		–	–	–		–	–
	Medicare	48 (29)	0.95		0.05–4.80	0.96				
	Medicaid	8 (5)	0.99		0.37–2.43	0.99				
	Other	15 (9)	0.84		0.13–3.03	0.81				
Charlson comorbidities	None	131 (80)	Ref		–	–	–		–	–
	1	24 (15)	1.13		0.32–3.06	0.83				
	≥ 2	8 (5)	1.78		0.28–6.22	0.47				
**Clinicopathologic characteristics**										
Site	Stomach	103 (63)	Ref		–	–	Ref		–	–
	Small intestine	60 (37)	2.40		1.05–5.62	0.038	2.72		1.13–6.69	0.026
Tumor size	≤ 5 cm	52 (32)	Ref		–	–	Ref		–	–
	> 5 cm	84 (52)	5.49		1.57–34.6	0.0050	3.42		0.90–22.5	0.073
	Tx	27 (17)	7.29		1.39–53.6	0.019	4.45		0.79–34.3	0.091
Mitoses	≤ 5 per 50 HPF	108 (66)	Ref		–	–	Ref		–	–
	> 5 per 50 HPF	36 (22)	5.62		2.26–15.1	< 0.001	4.77		1.86–13.2	0.0012
	Unspecified	19 (12)	4.05		1.06–13.5	0.042	3.59		0.92–12.2	0.064
Multifocality	Absent	121 (74)	Ref		–	–	–		–	–
	Present	30 (18)	1.15		0.38–2.92	0.78				
	Unspecified	12 (7)	0.66		0.04–3.21	0.67				
Node status	Negative	58 (36)	Ref		–	–	–		–	–
	Positive	11 (7)	0.95		0.15–3.55	0.95				
	Unknown	94 (58)	0.56		0.23–1.32	0.18				
Metastasis at diagnosis	Absent	144 (88)	Ref		–	–	–		–	–
	Present	19 (12)	0.81		0.13–2.76	0.77				
**Treatment characteristics**										
Margins	Negative	125 (77)	Ref		–	–	–		–	–
	Positive	10 (6)	2.87		0.66–8.81	0.14				
	Unknown	9 (6)	3.00		0.69–9.21	0.13				
	Not performed	19 (12)	1.76		0.41–5.40	0.40				
Systemic therapy	None given	77 (47)	Ref		–	–	Ref		–	–
	Given	84 (52)	3.15		1.25–9.60	0.014	1.66		0.63–5.27	0.32
	Unknown	2 (1)	5.83		0.30–36.3	0.19	1.83		0.09–13.4	0.62

*HPF* high-power field, *OR* odds ratio, *CI* confidence interval.

## Discussion

This study evaluated a large national cohort of patients with WT GIST to assess patient demographics, clinicopathologic features, treatment patterns, and outcomes. Our analyses found that most patients were male, middle-aged, and Caucasian. Primary tumors were often identified in the stomach, but about a third originated from the small intestine. Colorectal or esophageal primaries were rare. Most tumors were greater than 5 cm in maximum dimension yet had 5 or fewer mitoses per 50 HPF. Multifocality (17%), lymph node dissemination (7%), and synchronous metastatic disease at diagnosis (14%) were relatively uncommon. Most patients underwent surgical resection which largely achieved negative tumor margins. Of note, over half of patients received pre- or post-operative systemic therapy. When evaluating those patients with gastric or small intestinal primaries, small intestine origin and > 5 mitoses per 50 HPF were independently associated with decreased overall survival.

The current study provides additional evaluation of WT GIST in a national cohort, which provides the updated perspective that these rare tumors can occur in older patients, males, and arise from the small intestine at higher frequencies than previously recognized by Weldon and colleagues in detailing the outcomes of 76 patients at the NIH Pediatric and Wild-Type GIST Clinic^4^. Although analysis of the NIH patient cohort was very informative, it arguably did not provide a complete picture of the spectrum of WT GIST disease given that it was a selective population. Specific differences between the reported NIH cohort versus our current NCDB national cohort include median age of diagnosis (21 years vs. 59 years) and patient sex (75% vs. 45% female)^4^. Furthermore, we identified a greater proportion of small intestinal primaries, and this primary disease site was associated with worse overall survival on multivariable analysis. We also found that systemic therapy was commonly administered to patients; however, this was not associated with an overall survival benefit on multivariable analysis. This is consistent with limited efficacy of TKI therapy observed in this population and underlines the need for identification of improved systemic therapies for WT GIST.

Additionally, our results can be interpreted in context of existing national database analyses of patients with GIST. In an investigation using the SEER database and focusing specifically on younger patients with GIST, Fero and colleagues found that only 7% of patients with GIST overall were under the age of 40 years; within this population, most patients were male and aged 30–39 years^12^. The primary sites of disease were predominantly gastric, with a significant proportion of small intestinal origin (36%). A similar evaluation of the Dutch GIST registry noted that young adults comprised 5% of all patients, of whom 46% had gastric primaries, 46% had small bowel primaries, and 25% had non-*KIT*/*PDGFRA* mutations^13^. Furthermore, just over half (54%) of patients in this population were male. Our findings that 55% of patients with WT GIST in the NCDB were male and that a significant proportion had a small bowel primary are consistent with these other national database studies. In contrast to the SEER analysis, however, we found that when analyzing the NCDB WT GIST cohort, small bowel primary tumor location was associated with significantly worse OS. This poor outcome in small bowel disease is consistent with previous reports on small intestinal GIST^14^ and should alert clinicians regarding the aggressive nature of these tumors. Interestingly, a previous study by Boikos and colleagues had identified a subset of wildtype GIST patients with competent SDHB that had a predilection for older age and small bowel primary site. Our analysis confirms that existence of this patient population and highlights the need for comprehensive mutational analysis of small bowel GISTs to establish meaningful genotype–phenotype correlations that may improve outcomes in patients with these tumors^7^.

Limitations of this study include the lack of capture of specific non-*KIT/PDGFRA* somatic and/or germline mutations/epigenetic silencing of other drivers such as *SDHx* or *NF1,* as well as documentation of co-occurring mutations (other than *KIT/PDGFRA*). Indeed, advances in sequencing technologies have recently revealed that GIST mutational landscape and response to therapy can be influenced greatly by presence of synchronous oncogenic mutations, precise anatomic location within a specific organ, as well as expression levels of oncogenes^15–17^. Database studies such as the NCDB fails to comprehensively capture these details. Furthermore, while NCDB identifies patients undergoing systemic therapy, the precise identity of the agent (eg. cytotoxic chemotherapy vs. kinase inhibitors vs. metabolic agents) is not captured, thus limiting further inference regarding the utilization and impact of specific agents. The inability to assess DSS in addition to OS also remains an intrinsic limitation of any NCDB study. Nevertheless, the nature of NCDB case capture allowed for identification of an otherwise non-selected patient analysis, which complements and expands upon the patient population studied by Weldon and colleagues at the WT GIST Clinic.

In summary, we believe that this analysis provides a more complete representation of the spectrum of patients with WT GIST than is available from the NIH WT GIST cohort alone. Furthermore, the availability of specific data regarding *KIT/PDGFRA* mutation status, capture of surgical margin status, and far improved completeness of mitotic rate provides more robust data than possible through SEER. Our findings highlight important similarities and intriguing differences compared to the SEER and NIH cohorts, thereby adding to the current literature for non-*KIT/PDGFRA*-driven GIST disease. This study adds to the growing global literature on WT GIST disease to further dispel the misconception that patients with WT GIST are predominantly pediatric aged, female, and limited to the stomach. The importance of accurate and complete molecular characterization of GISTs among patients across the age spectrum is important to identify and most appropriately treat patients with wild-type *KIT* and *PDGFRA* GIST.

## Methods

The NCDB is a joint project of the Commission on Cancer of the American College of Surgeons and the American Cancer Society. Patients captured within the database represent seventy percent of those with new cancer diagnoses treated at the approximately 1500 Commission on Cancer (CoC) designated centers across the United States. Given the de-identified nature of the data, this study was exempted from institutional review board approval.

**Figure 2:**
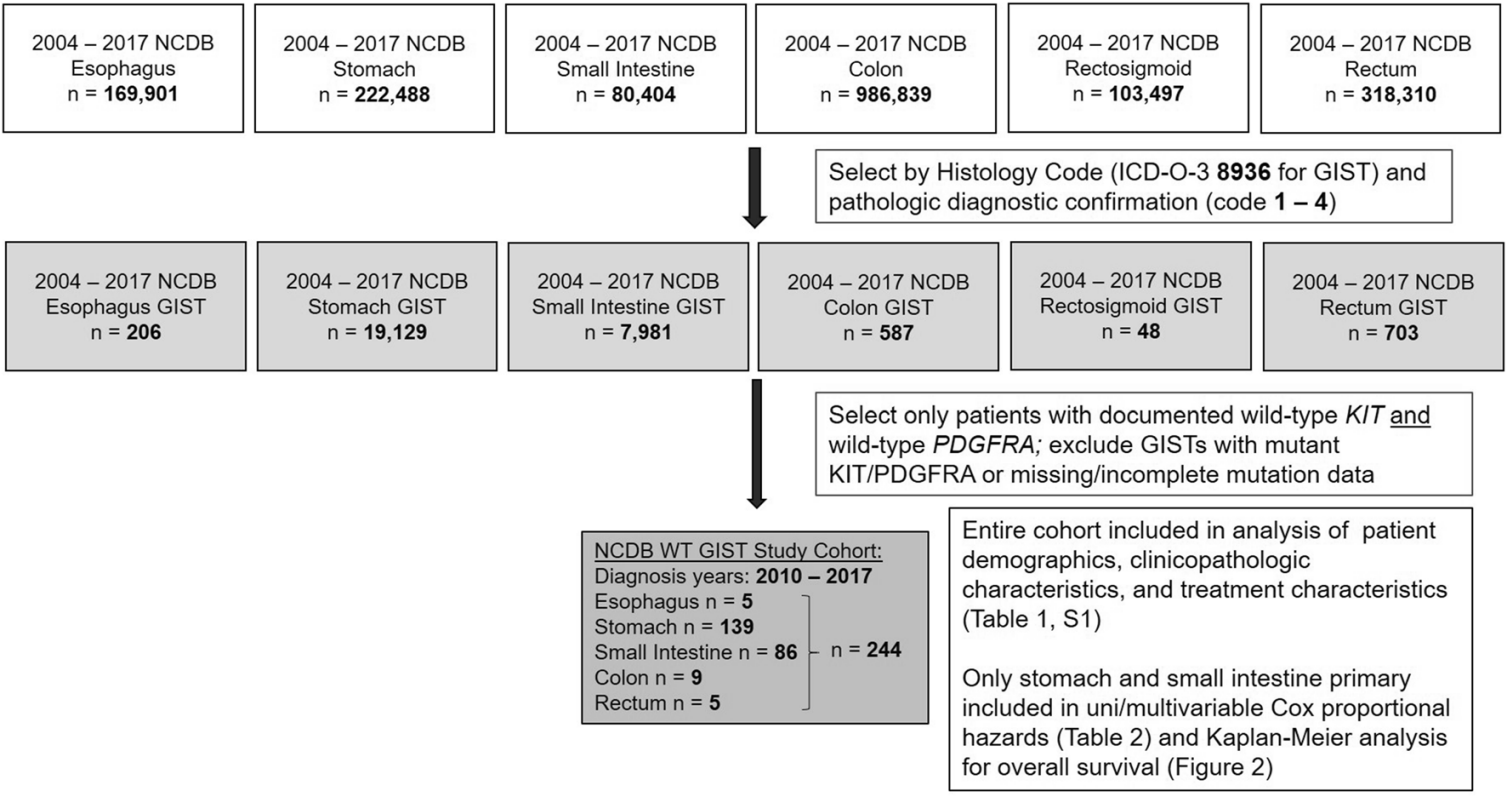
Flow diagram of patient selection from NCDB for this study.

Patient selection from the PUFs was performed as shown in Fig. 2. The 2004–2017 NCDB Participant User Files (PUFs) for the esophagus, stomach, small intestine, colon, rectosigmoid, and rectum were queried for all adult and pediatric patients with a diagnosis of GIST. Patients with GIST were identified based on International Classification of Diseases for Oncology, 3^rd^ Edition (ICD-O-3) morphology code 8936 with pathologic diagnostic confirmation. The NCDB has captured *KIT/PDGFRA* mutational status for GIST starting diagnosis year 2010, and patients with wildtype *KIT* and *PDGFRA* were identified based on the Site-Specific Factor (SSF) for *KIT* mutation status (esophagus/stomach/small intestine: SSF 8; colon/rectosigmoid/rectum: SSF 13) and *PDGFRA* mutation status (esophagus/stomach/small intestine: SSF 9; colon/rectosigmoid/rectum: SSF 14). Wild-type *KIT* status was indicated by SSF 8 or 13 value of 0, and wild-type *PDGFRA* status was specified by SSF 13 or 14 value of 20. Only patients with GISTs documented as being both *KIT* and *PDGFRA* wildtype were included in this analysis; GISTs harboring mutations or having incomplete/unknown mutations status were excluded entirely. Given mutational status has been captured in the NCDB since 2010, the final study population therefore included only patients diagnosed between 2010 and 2017.

Additional patient demographic, clinicopathologic, and treatment data captured included size, mitotic rate (esophagus/stomach/small intestine: SSF 6; colon/rectosigmoid/rectum: SSF 11), tumor multifocality (esophagus/stomach/small intestine: SSF 10; colon/rectosigmoid/rectum: SSF 15), and tumor location. Tumor size was stratified by ≤ 2 cm, 2.1 to 5 cm, and > 5 cm based on Armed Forces Institute of Pathology (AFIP) criteria and the nature of NCDB coding^18^. Mitotic rate was stratified as ≤ 5 per 50 high-powered field (HPF) at 40× or > 5 per 50 HPF. Tumor multifocality was stratified as absent, present, or unspecified. Location of gastric tumors was delineated by ICD-O-3 topographical codes, where proximal lesions were considered to be of the cardia (C160) and fundus (C161), body tumors to be of the body (C162), lesser curve (C165), or greater curve (C166), and distal lesions to be of the antrum (C163) or pylorus (C164); overlapping (C168) and not otherwise specified (C169) were grouped together. Location of small intestinal tumors was delineated as duodenal (C170), jejunoileal (C171, C172), or unspecified (C178, C179). Finally, we also included surgical margin status, extent of resection, presence of metastatic disease at the time of diagnosis, and systemic therapy administration. Patient tumor sequence number was limited to 0 or 1 only for survival analyses.

Data analysis was performed with Stata/MP software (version 16.0; StataCorp, College Station, TX) and JMP Pro (version 13.0.0, SAS Institute, Cary, NC). Statistical significance was defined as a two-tailed *P* value of < 0.05, or 95% confidence intervals (95% CI) that exclude 1.0, for all outcomes. Categorical variables were summarized using counts and percentages, with group differences assessed by Pearson’s *χ*^2^ test. Patients were censored at last follow-up date. Overall survival (OS) was modeled using the Kaplan–Meier method with log-rank testing to compare patient groups. Patient, disease, and treatment characteristics were evaluated with univariable and multivariable Cox proportional hazards analyses with OS as the outcome variable, expressed as hazards ratio (HR) and 95% CI. Only those characteristics associated with OS on univariable analysis were included for multivariable analysis. Survival analysis was limited to OS as NCDB does not provide data on disease-specific or recurrence-free survival.

## Supplementary Information

Supplementary Table S1.

## Data Availability

The data that support the findings of this study are available from American College of Surgeons, but restrictions apply to the availability of these data, which were used under license for the current study, and so are not publicly available. Data are however available from the authors upon reasonable request and with permission of American College of Surgeons.
