# Supplementary material for: Patient demographics, clinicopathologic features, and outcomes in wild-type gastrointestinal stromal tumor: a national cohort analysis
Source: Sci Rep. 2022 Apr 6;12:5774. doi: 10.1038/s41598-022-09745-1 (PMC8987058; doi:10.1038/s41598-022-09745-1)
Supplement: Supplementary file 1 — Supplementary Table S1. [file 41598_2022_9745_MOESM1_ESM.docx]

**Table Supplementary 1. Patient demographics, clinicopathologic characteristics, and treatment characteristics, including only stomach and small intestine primary site**

|  | | **Total**  **n = 225**  **N (%)** | **Stomach n = 139 N (%)** | **Small Intestine**  **n = 86**  **N (%)** | ***P* value** |
| --- | --- | --- | --- | --- | --- |
| **Patient Characteristics** | | | | | |
| Age at Diagnosis | < 18 | 8 (4) | 8 (6) | - | 0.09 |
|  | 18-40 | 34 (15) | 23 (17) | 11 (13) |  |
|  | 40-65 | 104 (46) | 59 (42) | 45 (52) |  |
|  | > 65 | 79 (35) | 49 (35) | 30 (35) |  |
| Sex | Male | 126 (56) | 77 (55) | 49 (57) | 0.82 |
|  | Female | 99 (44) | 62 (45) | 37 (43) |  |
| Race/Ethnicity | White | 159 (71) | 92 (66) | 67 (78) | 0.32 |
|  | Black | 25 (11) | 18 (13) | 7 (8) |  |
|  | Hispanic | 27 (12) | 19 (14) | 8 (9) |  |
|  | Other | 14 (6) | 10 (7) | 4 (5) |  |
| Insurance | Private | 115 (51) | 74 (53) | 41 (48) | 0.17 |
|  | Medicare | 78 (35) | 44 (32) | 34 (40) |  |
|  | Medicaid | 13 (6) | 6 (4) | 7 (8) |  |
|  | Other | 19 (8) | 15 (11) | 4 (5) |  |
| Charlson Comorbidities | None | 170 (76) | 109 (78) | 61 (71) | 0.26 |
|  | 1 | 41 (18) | 24 (17) | 17 (20) |  |
|  | ≥2 | 14 (6) | 6 (4) | 8 (9) |  |
| **Clinicopathologic Characteristics** | | | | | |
| Size | ≤2 cm | 11 (5) | 8 (6) | 3 (3) | 0.51 |
|  | 2.1 to 5 cm | 55 (24) | 31 (22) | 24 (28) |  |
|  | >5 cm | 97 (43) | 64 (46) | 33 (38) |  |
|  | Tx | 62 (28) | 36 (26) | 26 (30) |  |
| Grade | Low | 51 (23) | 29 (21) | 22 (26) | 0.77 |
|  | Moderate | 40 (18) | 24 (17) | 16 (19) |  |
|  | High | 17 (8) | 10 (7) | 7 (8) |  |
|  | Unknown | 117 (52) | 76 (55) | 41 (48) |  |
| Mitoses | ≤ 5 per 50 HPF | 150 (67) | 87 (63) | 63 (73) | 0.16 |
|  | > 5 per 50 HPF | 49 (22) | 32 (23) | 17 (20) |  |
|  | Not specified | 26 (12) | 20 (14) | 6 (7) |  |
| Lymph-vascular Invasion | Absent | 78 (35) | 51 (37) | 27 (31) | 0.25 |
|  | Present | 7 (3) | 6 (4) | 1 (1) |  |
|  | Unknown | 140 (62) | 82 (59) | 58 (67) |  |
| Multifocality | Absent | 167 (74) | 104 (75) | 63 (73) | 0.53 |
|  | Present | 38 (17) | 21 (15) | 17 (20) |  |
|  | Unspecified | 20 (9) | 14 (10) | 6 (7) |  |
| Node Status | Negative | 77 (34) | 42 (30) | 35 (41) | 0.13 |
|  | Positive | 15 (7) | 12 (9) | 3 (3) |  |
|  | Unknown | 133 (59) | 85 (61) | 48 (56) |  |
| Metastasis at Diagnosis | Absent | 192 (86) | 114 (83) | 78 (92) | 0.06 |
|  | Present | 31 (14) | 24 (17) | 7 (8) |  |
| **Treatment Characteristics** | | | | | |
| Surgical Margins | Negative | 163 (72) | 104 (75) | 59 (69) | **0.04** |
|  | Positive | 20 (9) | 7 (5) | 13 (15) |  |
|  | Unknown | 10 (4) | 5 (4) | 5 (6) |  |
|  | Surgery not performed | 32 (14) | 23 (17) | 9 (10) |  |
| Chemotherapy | No chemo given | 103 (46) | 66 (47) | 37 (43) | 0.51 |
|  | Chemo received | 119 (53) | 72 (52) | 47 (55) |  |
|  | Unknown | 3 (1) | 1 (1) | 2 (2) |  |
| HPF = high-power field | | | | | |
